# An Evaluation of an LCD Display With 240 Hz Frame Rate for Visual Psychophysics Experiments

**DOI:** 10.1177/2041669517736788

**Published:** 2017-10-16

**Authors:** Lin Shi

**Affiliations:** Faculty of Information Engineering and Automation, Kunming University of Science and Technology, China; Computer Application Key Laboratory of Yunnan Province, Kunming University of Science and Technology, China

**Keywords:** 240 Hz, LCD, display, visual psychophysics, color, time perception

## Abstract

Recently, a few LCD displays with 240 Hz frame rate have appeared on the market. I evaluated a LCD display with 240 Hz frame rate in terms of its temporal characteristics, progression between frames, and chromatic characteristics. The display showed (a) accurate frame durations at millisecond level, (b) gradual transition between adjacent frames, and (c) acceptable chromatic characteristics.

LCD technology is constantly improving in ways that are beneficial to the needs of visual scientists. It used to be that LCD displays were not suitable for displaying objects in high-speed motion because of relatively slow updating speeds of LCD pixels. Recently, a few LCD displays with 240 Hz frame rate have appeared on the market, designed for computer game players. Here, I evaluate a LCD display (ROG PG258Q, ASUS) with 240 Hz frame rate in terms of its temporal characteristics, progression between frames, and chromatic characteristics. The display showed accurate frame durations at millisecond level and a gradual transition between adjacent frames, with no evidence of ghosting from one frame to the next. The full response spectra for the three color channels are also presented.

Stimuli were controlled by web pages using HTML, JavaScript, and WebGL technology on a PC (Intel Core i7-6700 CPU, NVidia GeForce GTX 1060 GPU). The LCD display was connected to the graphics card through a DisplayPort cable of a PC running 64-bit Microsoft Windows 10 Home Edition (Chinese version). The web browser was Firefox (version number 53.0, 64-bit). OpenGL shader programs were used to make sure the stimuli were generated in real-time on the GPU and to make sure the stimuli were displayed reliably at the 240 Hz frame rate. The driver of the LCD display was set to a 1920 × 1080 resolution, 240 Hz frame rate, 32-bit color mode, and G-Sync was turned on (G-Sync is a sync technology developed by Nvidia aimed to eliminate screen tearing, and more details are available on the Nvidia web site).

## Temporal Characteristics

Temporal characteristics were measured by a light meter (LM03, Cambridge Research Systems Ltd., which is a demonstration product, provided freely at Asia Pacific Conference on Vision 2014, but not available for sale by CRS Ltd.) and a high-speed video camera (RX100-V, SONY). The LM03 light meter was designed for high temporal resolution but not necessarily accurate measurement of intensity. Precise measurement of output intensities to measure the gamma properties, for instance, is not the aim of this review. Temporal intensity variations of red, green, and blue channels and frame switching were measured as described later.

### Temporal Intensity Variations of Red, Green, and Blue Channels

**Figure 1.**
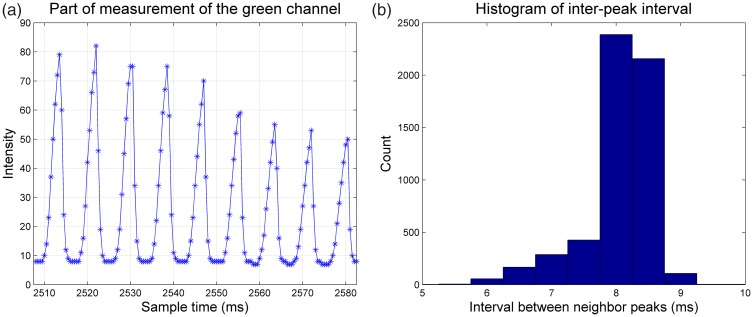
Temporal intensity variations. Pane A shows a section of temporal intensity measurements while frames alternated green and black frame, with a decreasing intensity. Measurements of red and blue frames were similar with those of green frames. The *x*-axis shows the sample time and the *y*-axis shows the intensity measured by the light meter in arbitrary units. Pane B shows the histogram of inter-peak interval which indicated most intervals between adjacent peaks were 8.0 to 8.5 ms.

A sequence of frames was presented, with red, green, and blue separately increasing (in 240 steps) from 0 to 1 and then decreasing again, with a black frame (r = 0, g = 0, b = 0) inserted between two continuous frames in order to separate continuous change clearly. The sample interval of the light meter was set as 500 microseconds and the monitor frame rate was set to 240 Hz. A subset of the data from the green channel measurements can be seen in Figure 1, which shows (a) the temporal intensity variation between the minimum value and the peak value was regular, sharp, and rapid; (b) transitions from dark-to-light were slower than transitions from light-to-dark; and (c) distance between adjacent peaks were in the range 8 to 8.5 ms which was consistent with frame duration 8.3 ms (two frames in 240 Hz frame rate). The variability may derive from aliasing between the sampling frequency (500 Hz) and the display frequency (240 Hz) rather than any actual imprecision in the frame rate.

### Frame Switching Observed by a High-Speed Camera

Animated numbers were displayed on a constant changing background. The number increased one at each frame. The frame rate was set as 240 Hz. The frame sequence was recorded by the high-speed video camera with a sample rate 1000 Hz.

**Figure 2.**
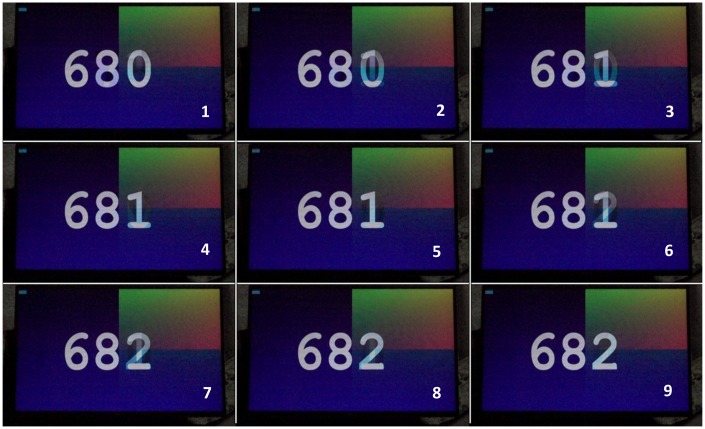
Continuous frames captured by the camera (sampling at 1 kHz) with the monitor updating at 240 Hz. Each displayed frame showed the frame number (680–682 here). Camera frames (numbered 1–9 here) showed the smooth transition from one display frame to the next, taking four frames to change, as expected. In camera Frames 1, 5, and 9, you can see that the frame has completely switched, with no evidence of ghosting from the previous frame.

Figure 2 shows nine continuous frames captured by the camera, representing three display frames on the monitor (labeled 680, 681, and 682). Display Frame 681 corresponded to camera Frames 2, 3, 4, and 5 and display Frame 682 corresponded to camera Frames 6, 7, 8, and 9. Camera Frames 2, 3, 4, 6, 7, and 8 showed the gradual changes between two display frames as the LCD pixels switched.

**Figure 3.**
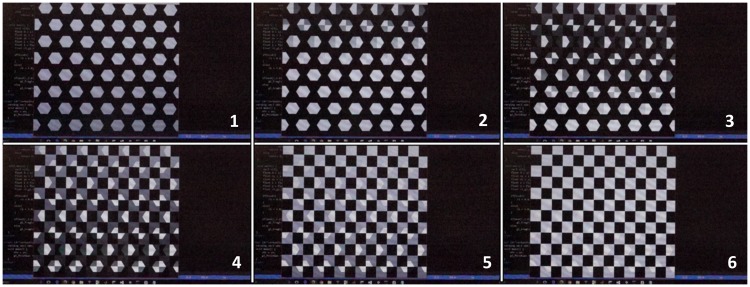
Frame switching between hexagons and checkerboard. From left to right and from top to down, there were six continuous camera frames (1–6). Frame 1 corresponded with a hexagon display frame and frames from 2 to 6 show the transition to a checkerboard display frame. There was fusion between the hexagons and the squares in frames from 2 to 5.

In Figure 3, we show the results of switching a larger pattern on the screen, with two adjacent frames showing a hexagonal checkerboard (camera Frame 1) and square checkerboard (camera Frame 6). Fusion between the hexagon and the square patterns was observed in white hexagons and white squares. Intermediate camera frames show the gradual transition between the images.

## Chromatic Display Characteristics

**Figure 4.**
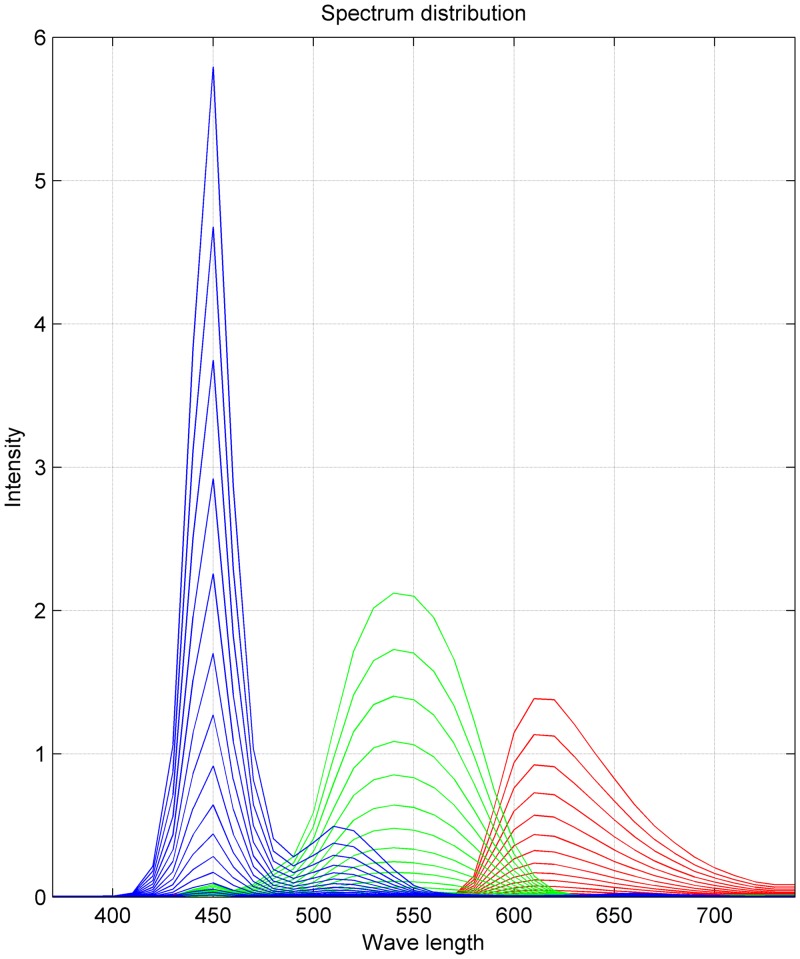
Spectrum of red, green, and blue channels. The r, g, and b values were varied from 0 to 1 in 16 steps separately and are shown as increasing lines in their corresponding color.

The stimulus frames were full screen uniform background which r, g, b values varied from 0 to 1 in 16 steps separately. A spectrometer (eye-one pro, X-rite) measured spectrum of the display from 390 nm to 740 nm with 5 nm step, as shown in Figure 4. Further testing is needed to determine the monitor’s chromatic characteristics precisely.

## Conclusion

This 240 Hz LCD display has the following characteristics: (a) accurate frame durations at millisecond level; (b) gradual transition between adjacent frames; and (c) acceptable chromatic characteristics.

